# Altered expression levels of occludin, claudin-1 and myosin light chain kinase in the common bile duct of pediatric patients with pancreaticobiliary maljunction

**DOI:** 10.1186/s12876-016-0416-5

**Published:** 2016-01-16

**Authors:** Shun-gen Huang, Wan-liang Guo, Zhi-cheng Zhou, Jun-jie Li, Fu-bin Yang, Jian Wang

**Affiliations:** Departments of Pediatric General Surgery, Children’s Hospital affiliated to Soochow University, Suzhou, Jiangsu 215003 China; Department of Radiology, Children’s Hospital affiliated to Soochow University, Suzhou, Jiangsu 215003 China

**Keywords:** Occludin, Claudin-1, Myosin light chain kinase, Pancreaticobiliary maljunction

## Abstract

**Background:**

In pancreaticobiliary maljunction (PBM), the sphincter of Oddi can not control bile and pancreatic juice flow, which may lead to two-way reflux of bile and pancreatic juice, thus causing chronic inflammation, thickening, fibrosis and metaplasia of the common bile duct wall. These pathophysiological changes have been linked to disruption of the epithelium barrier in the common bile duct. We hypothesized that the expression of tight junction-associated proteins may be dysregulated in the common bile duct in PBM. In the current study, we sought to analyze the expression of tight junction-associated proteins in the common bile duct epithelium of pediatric patients with PBM.

**Methods:**

Specimens of the common bile duct were collected from 12 pediatric patients with PBM and 10 non-PBM controls. The expression of the tight junction-associated proteins occludin and claudin-1 in the epithelium was examined by immunohistochemistry. The Image-Pro Plus v. 6.0 image analysis software was used to calculate the mean qualifying score (MQS) of imunostained sections of common bile duct epithelium. Total protein extracts of common bile duct were analyzed by Western blotting assays to examine expression of occludin, claudin-1 and myosin light chain kinase (MLCK). Spearman correlation analysis was used to analyze the relation between MLCK and occludin, MLCK and claudin-1.

**Results:**

Immunostained sections of the common bile duct epithelium showed significantly higher MQS in pediatric patients than controls for occludin (44.11 ± 13.82 vs. 11.30 ± 9.58, *P* = 0.0034) and claudin-1 (63.44 ± 23.59 vs. 46.10 ± 7.84, *P* = 0.0384). Western blotting also showed significantly higher expression of occludin, claudin-1 and MLCK in the common bile duct of patients than of controls (*P* = 0.0023, 0.0015, 0.0488). Spearman correlation analysis showed that MLCK expression correlated positively with the expression of occludin (*r*_s_ = 0.61538, *P* = 0.0032) and claudin-1 (*r*_s_ = 0.7972, *P* = 0.0019).

**Conclusions:**

Occludin and claudin-1 are up-regulated in the common bile duct epithelium of pediatric PBM patients. MLCK may be involved in the process of up-regulation of the tight junction-associated proteins in PBM.

## Background

Pediatric pancreaticobiliary maljunction (PBM) is a rare congenital anomaly in which the main pancreatic and bile ducts are joined outside the duodenal wall and form a long common channel. The sphincter of Oddi in PBM can not control bile and pancreatic juice flow, which may lead to two-way reflux of bile and pancreatic juice [1]. Pancreatic juice regurgitation causes mixing of the pancreatic juice and bile, which activates multiple pancreatins in the biliary duct, including phospholipase A2 and trypsin, leading to chronic inflammation of the biliary duct wall. Chronic inflammation of the biliary duct eventually destroys the epithelial barrier and up-regulates the expression of proliferating cell nuclear antigen (PCNA), p53 and K-ras in biliary epithelial cells, leading to malignant transformation [2–5].

Little is understood about how the epithelial barrier in the common biliary duct is destroyed in patients with PBM. A molecular understanding of this process may help develop diagnostic markers for early detection of PBM-related complications, including cancer, as well as identify proteins and signaling pathways that may be suitable targets for therapy.

Tight junctions are selectively permeable areas where the membranes of neighboring cells lie in close apposition, forming a virtually impermeable barrier to fluid and maintaining cell polarity [6–8]. They are formed by heterogeneous protein complexes containing numerous proteins, including occludin, cingulin, symplekin, junctional adhesion molecules, and members of the claudin and zonula occludens (ZO) protein families [9, 10]. The integrity of the epithelial barrier in the common biliary duct depends on tight junctions between biliary duct epithelial cells, which form an epithelial barrier that prevents toxic substances, inflammatory factors and pathogenic microorganisms from entering the bile duct [11–16]. Occludin localizes specifically to tight junctions, and its phosphorylation helps ensure correct localization of functional junction complexes. Thus, occludin is critical for maintaining intercellular permeability and transepithelial resistance [6]. Each of the various claudins in tight junctions has a particular ion selectivity for ion transfer across the barrier [7, 8]. The precise claudin composition in tight junctions varies across tissues. For example, claudin-1 is important for maintaining the gastrointestinal mucosal barrier [17]. Both occludin and claudin-1 play key roles in the biliary epithelial barrier; in humans, and they localize primarily to the bile duct and bile canaliculus [18].

It is possible that epithelial barrier disruption in PBM involves tight junction dysregulation. Consistent with this idea, experiments with a cell culture model system of IgG4-related cholangitis point to dysfunction of the biliary epithelial cell barrier [19]. In addition, experiments showed that disruption of tight junction function and subsequent leakage of the bile constituents may influence the aggravation of cholestasis in primary biliary cirrhosis [20].

Fallon *et al.* reported that the levels of ZO-1 progressively increased to 3-fold the levels in controls by 9 days after induction of extrahepatic cholestasis; the levels of occludin decreased within 2 days, which then gradually recovered to the control levels by 9 days [11, 21]. Takakuwa et al. reported the mRNA transcript levels of *occludin* increased in rat livers at 6 h after the common bile duct was ligated [12]. These studies suggest that disorders of the bile duct are associated with altered expression of tight junction-associated proteins, raising the possibility that the expression of tight junction-associated proteins may be altered in PBM.

To examine whether PBM disrupts the epithelial cell barrier and identify possible molecular pathways involved, we measured the levels of several tight junction-associated proteins in common bile duct tissue specimens from pediatric patients with PBM. We focused on occludin, claudin-1 and myosin light chain kinase (MLCK), which have been shown to destroy epithelial barrier function [22, 23]. The results lay the foundation for future studies to understand the cause of epithelial barrier disruption.

## Methods

### Subjects

The study protocol was approved by the Institutional Ethics Review Committee at Children’s Hospital affiliated to Soochow University and the supervising local health ministry. Informed consent was obtained from the legal surrogates of the subjects following a detailed description of the purpose of the study. All experiments were carried out in strict accordance with the institution guidelines regarding the acquisition and experimental use of human tissues. Data were retrospectively analyzed for all 12 pediatric patients (7 boys; median age, 3 years; age range, 5 months – 8 years) diagnosed with PBM who were admitted to our hospital between January 2011 and December 2014. In all patients, diagnosis was confirmed by imaging and surgical pathological examination. PBM was diagnosed based on the following criteria: (1) the union of pancreatic and biliary duct was located outside the sphincter of Oddi, based on magnetic resonance cholangiopancreatography (MRCP) or intraoperative cholangiography (IOC); (2) the common duct was longer than 5 mm; (3) the biliary amylase level was greater than 1000 U/L [24, 25]. Common bile duct specimens were taken from PBM patients and stored −20 °C until use.

As non-PBM controls, common bile duct specimens were also collected from 10 deceased neonatal or pediatric patients (6 boys; median age, 5 days; range, 1 day to 8 years). The causes of death included neonatal respiratory distress syndrome (n = 3), neonatal septicemia (n = 2), neonatal pulmonary hemorrhage (n = 2), intracranial hemorrhage (n = 2), and complex congenital heart disease (n = 1). Pancreaticobiliary disease was ruled out in all controls.

### Immunohistochemistry

Paraffin-embedded specimens of the common bile duct were serially sectioned (5 μm thick). Sections were dewaxed and incubated in 3 % H_2_O_2_ at room temperature for 30 min to inactivate endogenous peroxidase. After rinse with distilled water and soak in phosphate-buffered saline (PBS), antigen retrieval was done with citrate buffer in a microwave oven, followed by blocking with 10 % goat serum. Sections were then incubated with anti-occludin antibody (1:300; Abcam, Cambridge, UK) and anti-claudin-1 antibody (1:250; Abcam) at 4 °C overnight and then at 37 °C for 45 min. Subsequently, the sections were incubated with biotin-conjugated secondary antibody, transferred to a 37 °C water bath for 20 min, incubated with horseradish peroxidase (HRP)-conjugated streptavidin, placed in a 37 °C water bath for 20 min, and rinsed with PBS four times (5 min each time). The sections were visualized with DAB, re-stained with hematoxylin, dehydrated, made transparent and enveloped. In parallel, positive controls (Abcam) were processed and all gave positive staining; negative controls were performed by replacing the primary antibody with PBS, and all were negative for staining.

Sections containing epithelial tissue that stained positive for occludin and claudin-1 were observed under a laser confocal scanning microscope (BX50 Olympus, Japan) at a magnification of 400 ×. Immunohistochemical staining was quantitated using IPP 6.0 image analysis software (Media Cybernetics, USA), and 5–8 fields of view were selected on each section and photographed. Image analyses were performed as described [26], and qualifying scores (QS) were calculated using the following formula: QS = percentage of positive cells × mean intensity. Mean QS (MQS) were obtained for the various fields of view in each section.

### Western blotting assays

Common bile duct specimens were weighed, and 100-mg samples were placed in 1 mL RIPA extraction buffer and 10 μL phenylmethanesulfonyl fluoride (PMSF), ground up, and centrifuged at 17,226 g for 30 min. The supernatant was collected and stored at −20 °C until use. Protein concentration was detected using the BCA method (Pierce, USA). SDS-PAGE was performed using a 5 % stacking gel and 15 % separation gel. Target protein bands were transferred to a nitrocellulose membrane, blocked with non-specific antibody, incubated overnight at 4 °C with anti-occludin antibody (1:250; Abcam), anti-claudin-1 antibody (1:200; Abcam), and anti-MLCK antibody (1:400; Abcam). After incubation with HRP-conjugated secondary antibody, the protein bands were visualized with enhanced chemiluminescence. Band intensities were quantified using digital imaging analysis software (Tanon-1600, China).

### Statistical analysis

All measurements were expressed as mean ± standard deviation (SD). Differences in MQS (immunohistochemistry) or band intensities (Western blotting) between patients and controls were tested for statistical significance using Student’s t test if they showed a normal distribution; otherwise, they were tested using Wilcoxon test. Possible correlation of MLCK band intensities with occludin or claudin-1 band intensities was assessed using Spearman rank correlation analysis. *P* < 0.05 was considered the threshold of significance.

## Results

### Immunohistochemistry of tight junction-associated proteins

**Fig. 1:**
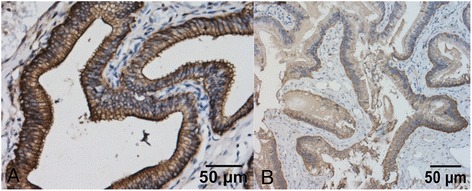
Claudin-1 is expressed primarily on the membrane of epithelial cells and less strongly in the cytoplasm and nucleus (**a**: PBM, **b**: control)

The epithelial cells of the common bile duct appeared columnar, and claudin-1 was expressed primarily on the membrane of epithelial cells and less strongly in the cytoplasm and nucleus (Fig. 1). The MQS for claudin-1 in the epithelial cells of the common bile duct were 63.44 ± 23.59 for PBM patients and 46.10 ± 7.84 for controls (P = 0.0384).

**Fig. 2:**
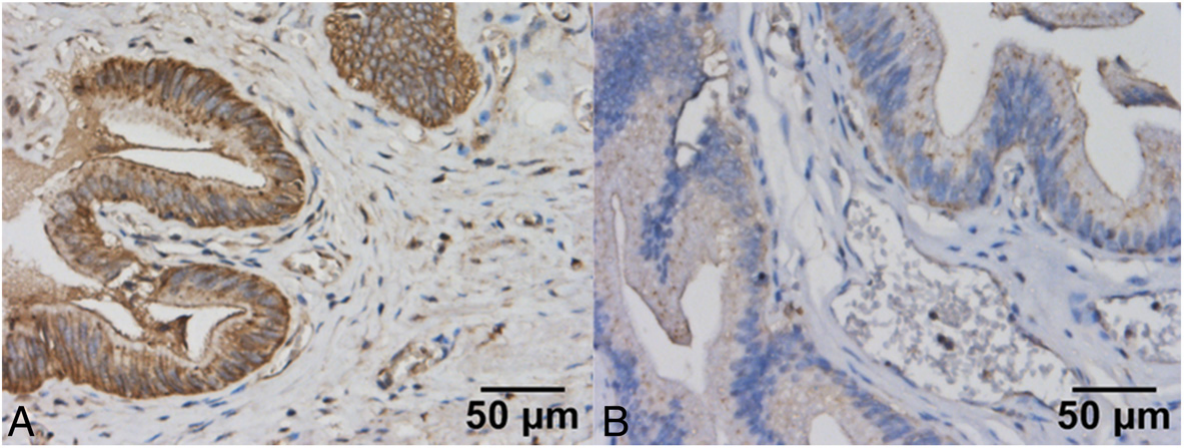
Occludin is expressed at moderate levels on the membrane of epithelial cells, and at very low levels in the cytoplasm and nucleus (**a**: PBM, **b**: control)

Occludin was expressed at moderate levels on the membrane of epithelial cells, and scantly in the cytoplasm and nucleus (Fig. 2). The MQS for occludin in the epithelial cells of the common bile duct were 44.11 ± 13.82 for PBM patients and 11.30 ± 9.58 for controls (*P* = 0.034; Table 1).

**Table 1:** Immunohistochemical analysis of occludin and claudin-1 expression in the epithelial cells of the common bile duct of pediatric patients with pancreaticobiliary maljunction (PBM) and non-PBM controls

Group		N	Mean ± SD^a^	P**
occludin	PBM	12	44.11 ± 13.82	0.0034
	Control	10	11.30 ± 9.58	
claudin-1	PBM	12	63.44 ± 23.59	0.0384
	Control	10	46.10 ± 7.84	

^a^Mean qualifying scores

**Wilcoxon two-sample test

### Western blotting of tight-junction associated proteins

**Fig. 3:**
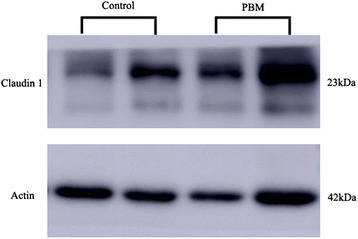
Representative Western blot of claudin-1 in the PBM group versus the control group

**Fig. 4:**
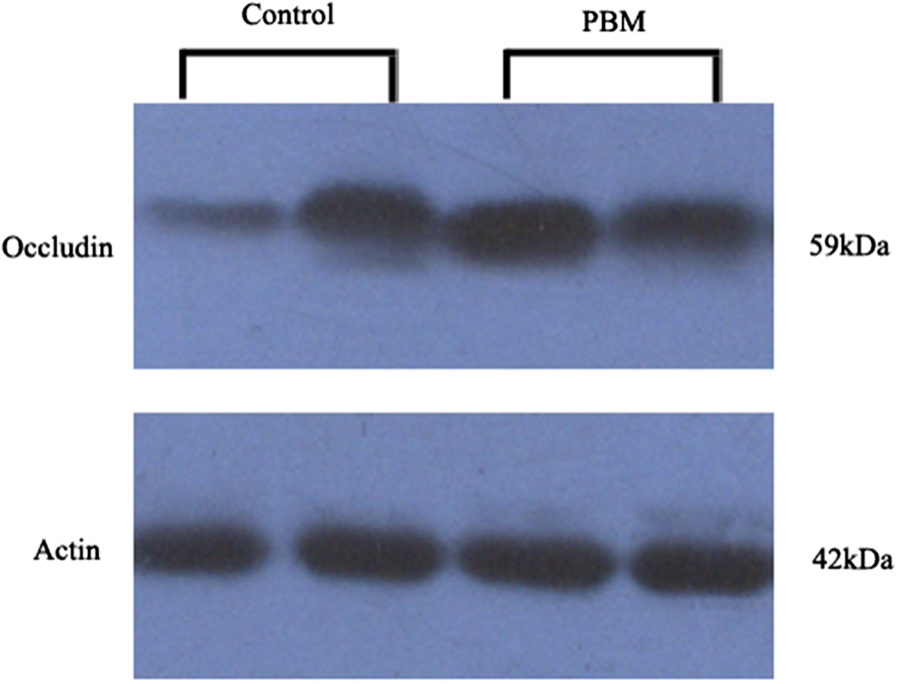
Representative Western blot of occludin in the PBM group versus the control group

**Fig. 5:**
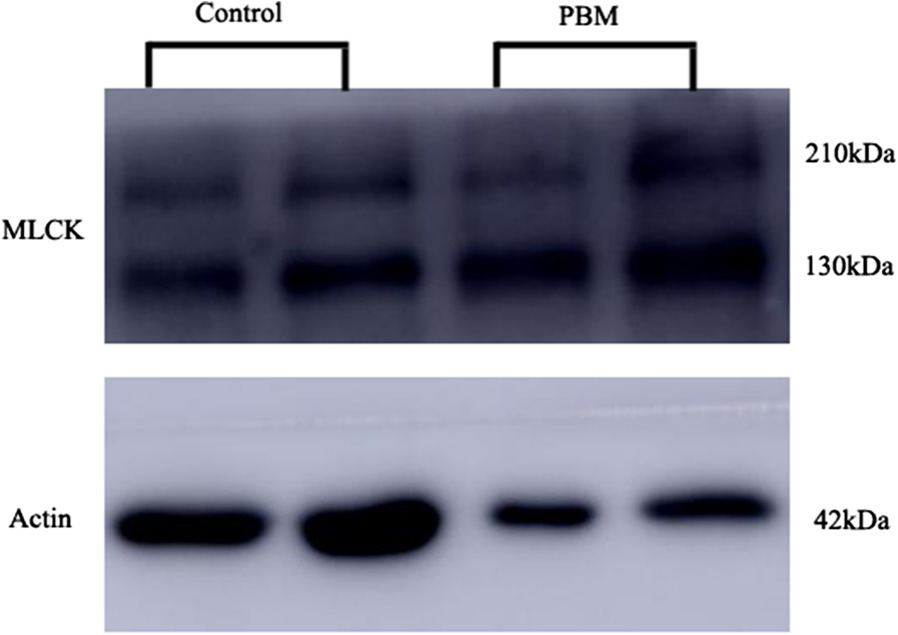
Representative Western blot of MLCK in the PBM group versus the control group

Western blotting assays of total protein extracts from common bile duct tissue showed significantly higher levels of the three tight junction-associated proteins in PBM patients than in controls (Table 2): claudin-1, 0.77 ± 0.54 vs. 0.20 ± 0.19 (*P* = 0.0023) (Fig. 3); occludin, 0.67 ± 0.46 vs. 0.12 ± 0.10 (P = 0.0015) (Fig. 4) and MLCK, 0.69 ± 0.58 vs. 0.25 ± 0.10 (P = 0.0488) (Fig. 5). Spearman rank correlation analysis revealed that MLCK expression correlated positively with the expression of both occludin (r = 0.61538, *P* = 0.0032) and claudin-1 (r = 0.7972, P = 0.0019) (Table 3).

**Table 2:** Expression of occludin, claudin-1 and myosin light chain kinase (MLCK) in the common bile duct of pediatric patients with PBM and non-PBM controls

Group		N	Mean ± SD	P**
MLCK	PBM	12	0.69 ± 0.58	0.0488
	Control	10	0.25 ± 0.10	
Occludin	PBM	12	0.67 ± 0.46	0.0015
	Control	10	0.12 ± 0.10	
Claudin-1	PBM	12	0.77 ± 0.54	0.0023
	Control	10	0.20 ± 0.19	

**Wilcoxon two-sample test

**Table 3:** Correlation between expression of MLCK protein and expression of tight junction-associated proteins in the common bile duct

Tight junction protein	Spearman rank correlation	
	*r*_s_	*P*
Claudin-1	0.79720	0.0019
Occludin	0.61538	0.0032

## Discussion

Here we showed that the expression of the tight junction-associated proteins occludin and claudin-1 is up-regulated in the epithelial cells of the common bile duct of PBM patients, which provides the first molecular clue that may help explain how this anatomical anomaly leads to disruption of the epithelial barrier in the common bile duct. We further demonstrated that the expression of MLCK, which may be involved in the disruption of the epithelial barrier in PBM, was up-regulated in the epithelial cells of the common bile duct of PBM patients.

In PBM, chronic contact between regurgitated pancreatic juice and the epithelial tissue of the common bile duct leads to chronic inflammation, thickening, fibrosis and metaplasia of the common bile duct wall [27]. The fibrosis further causes uneven bile discharge as well as elevated pressure and dilation within the common bile duct [28]. These pathophysiological changes have been linked to disruption of tight junctions in bile duct disorders [29] and cholestasis, which shows similar pathology to the cystic dilation of the common bile duct present in all our patients. In a rat model of cholestasis, the severity of changes in epithelial morphology and permeability in the common bile duct correlated with impairment of junctional integrity [30]. Ligating the common bile duct in rats caused a redistribution of occludin [11, 21]; the same procedure also up-regulated the mRNA transcript levels of occludin without affecting the mRNA transcript levels of claudin-1 or claudin-2 [12]. These findings demonstrate that cholestasis affects the expression and distribution of tight junction-associated proteins, which may help explain our results in PBM. At the same time, our observation that claudin-1 was up-regulated in the epithelial cells of the common bile duct of PBM patients suggests that cholestasis alone may not explain all our findings. The up-regulation of the tight junction-associated proteins occluding and claudin-1 is mainly attributable to the reflux of pancreatic juice into common bile duct and cholestasis in PBM. This may represent an adaptational response to preserve barrier function.

The up-regulation of occludin and claudin-1 in our patients with PBM correlated with expression of MLCK, and MLCK levels were significantly higher in patient tissues than in control tissues. These results are consistent with our previous work showing elevated MLCK expression in the common bile duct of patients with PBM [31]. This kinase helps to regulate the dynamic structure and function of tight junctions, and it may be activated by factors that damage the epithelial barrier [32, 33]. Our findings prompt us to speculate that MLCK dysregulation may be associated with impairment of the epithelial barrier in the common bile duct in patients with PBM, which, however, awaits further investigations.

Up-regulation of MLCK expression may compromise the epithelial barrier via several possible mechanisms. It may lead to increased myosin phosphorylation as alterations of epithelial contraction have been shown to increase mucosal permeability [33]. In intestinal epithelial disorders caused by inflammation or endotoxin, alteration of MLCK expression may alter the expression levels of tight junction-associated proteins [22, 23, 34]. MLCK has already been implicated in processes that damage the epithelial barrier in the bile duct, at least in tight junction destruction induced by lipopolysaccharide or H_2_O_2_: this damage is blocked by specific MLCK inhibitor ML-7 [35, 36]. Oxidative stress damage, which has been shown to increase MLCK activity [37], is also present in patients with PBM.

This study has several limitations. First, it was a retrospective and observational study, and therefore there may have been some selection bias. Second, some of the controls were from neonates. Neonatal tissues are special and different from tissues of persons of older ages, which may cause some selection bias. Third, the sample size is small. Therefore, further animal experiments and prospective studies are needed.

## Conclusions

In summary, our data show that PBM in pediatric patients is associated with up-regulation of the tight junction-associated proteins occludin and claudin-1 in the common bile duct epithelium. Furthermore, the expression of these two proteins with PBM correlated with that of MLCK, which may be involved in the process of up-regulation of the tight junction-associated proteins in PBM. Our findings pave the way for future studies to examine these alterations in real time, in the presence of MLCK inhibitor ML-7 and while measuring transepithelial resistance. Such work may help identify biomarkers for early detection and diagnosis of PBM-related complications, as well as elucidate pathological pathways that can be targeted in drug development.
